# Using Meta-Analysis and Propensity Score Methods to Assess Treatment Effects Toward Evidence-Based Practice in Extensive Reading

**DOI:** 10.3389/fpsyg.2020.00617

**Published:** 2020-04-22

**Authors:** Akira Hamada

**Affiliations:** Department of English, Faculty of Languages and Cultures, Meikai University, Urayasu, Japan

**Keywords:** evidence-based practice, quantitative methods, treatment effect assessment, meta-analysis, propensity score analysis, extensive reading

## Abstract

This study aimed to depict the assessment process of treatment effects of extensive reading in a second language (L2) toward the establishment of an evidence-based practice. Although standardized mean differences between treatment and control groups have been applied to interpret the magnitude of treatment effects in observational studies on L2 teaching, individual effect sizes vary according to differences in learners, measures, teaching approaches, and research quality. Prior research on extensive reading has suffered from methodological restrictions, especially due to a lack of appropriate comparison between treatment and control groups. For these reasons, a retrospective meta-analysis including only studies that ensured between-group equivalence was conducted in Study 1 to estimate the effect sizes of extensive reading expected in specific teaching environments. When the focused skill of the one-semester program was reading comprehension, its effect size was predicted as *d* = 0.55. However, the moderator analysis showed that this treatment effect was overestimated due to selection bias in the analyzed studies and adjusted the effect size from 0.55 to 0.37. In Study 2, propensity score analysis was applied to minimize selection bias attributed to observed confounding variables in the comparison between non-randomized treatment and control groups. Data were collected from 109 Japanese university students of English who received in-class extensive reading for one semester and 115 students who attended another English class as the control group. Various types of matching were attempted, and in consideration of balancing the five covariates that might affect treatment effect estimation, the best solutions were nearest neighborhood matching without replacement, nearest neighborhood matching with replacement, and full matching. The results showed that the average treatment effects of extensive reading on all the participants (*d* = 0.24–0.44) and on the treated individuals (*d* = 0.32–0.40) were both consistent with the benchmark established in Study 1. Pedagogical implications and methodological limitations are discussed for decision-making regarding the implementation of L2 teaching practices based on research evidence.

## Introduction

Treatment effect assessment in second language (L2) teaching plays an important role in determining its efficacy and utility and in facilitating pedagogical decision-making. Theories and hypotheses of L2 pedagogy have been proposed based on the variety of scientific evidence available in this field. Regarding this evidence, L2 teaching research has reported that effect sizes consist of the magnitude of treatment effects estimated by comparing treatment and control groups (e.g., Mackey and Gass, 2015; Marsden et al., 2018a). However, effect sizes from individual studies are not always applicable to other cases for pedagogical decision-making because of differences in research quality (Lipsey and Wilson, 1993; Plonsky and Gass, 2011; Plonsky and Oswald, 2014). An additional factor is the differences in study conditions, including participants, measures, and teaching approaches (Norris and Ortega, 2000). Given that a practical concern of L2 teaching is determining the type of instruction most applicable to a given class (Sato and Loewen, 2019), it is essential that treatment effect assessment provide information that facilitates effective pedagogical decision-making.

The concept of evidence-based practice provides a useful reference for pedagogical decision-making. In evidence-based practice, evidence is graded based on the quality of individual studies’ research design, validity, and applicability (Chambless and Ollendick, 2001). The present study, therefore, aimed to establish a system of treatment effect assessment founded on evidence-based practices regarding the use of extensive reading for teaching L2 reading. The treatment effect of extensive reading has been reproduced several times (Day, 2015; Waring and McLean, 2015; Yamashita, 2015) and has been synthesized as available research evidence by two meta-analyses (Nakanishi, 2015; Jeon and Day, 2016). However, prior studies on extensive reading have been problematic due to deficits in measurements (Al-Homoud and Schmitt, 2009; Beglar et al., 2012), design, and analysis (Nakanishi, 2015; Suk, 2017). To argue whether extensive reading is an evidence-based approach to teaching L2 reading, it is necessary to introduce improved methodologies for accurate assessment of its treatment effects.

## Literature Review

### Meta-Analysis for Evidence-Based Practice

Since the start of the movement toward medical evidence-based practice in the early 1990s, evidence-based practice has spread across intervention studies in psychology as well as in education. The APA Presidential Task Force on Evidence-Based Practice (2006) described it as the integration of the best research evidence with practitioners’ expertise in making decisions about interventions for individuals. In applied linguistics, the concept has been interwoven with policy-level educational decision-making (Pachler, 2003). For example, Mitchell (2000) suggested that L2 researchers would be required to offer an interpretation of current research evidence while engaging in ongoing policy debates. More recently, Sato and Loewen (2019) discussed evidence-based L2 pedagogy from the perspective of transferability of L2 acquisition research for classroom-level pedagogical decision-making. This is consistent with the core idea of evidence-based practice in psychology: to make practical interventions more effective by applying empirically supported principles of treatments (Chambless and Ollendick, 2001).

Evidence-based practice starts by determining which research evidence will assist individuals in achieving the best outcome. According to the APA Presidential Task Force on Evidence-Based Practice (2006), any practical intervention should be evaluated in terms of its efficacy and utility. Efficacy refers to the strength of research evidence for determining causal relationships between treatments and outcomes. Utility indicates the feasibility of treatments, including generalizability, acceptability of participants, costs, and benefits. Efficacy and utility are accepted as the basis of practical significance in L2 teaching research (Plonsky and Oswald, 2014). For example, evidence-based L2 pedagogy as proposed by Sato and Loewen (2019) emphasizes the importance of L2 teaching utility. To this end, they recommended using a quasi-experimental design to balance ecological validity and internal/external research validity to maximize the transferability of L2 research findings to classroom conditions.

Although multiple types of research evidence evaluate the efficacy and utility of interventions, pedagogical decisions should be made by considering a hierarchy of research evidence quality. Table 1 summarizes the levels of evidence for interventions, developed by the Oxford Centre for Evidence-Based Medicine (2009). When addressing a research question such as, “Does this intervention help?” the highest quality evidence is the expected treatment effects obtained through a systematic review of the research outcomes of randomized controlled trials (Level 1a). L2 teaching research has also evaluated treatment effects and intervention utility from synthesized research outcomes considering factors such as differences in populations, interventions, and settings (e.g., Norris and Ortega, 2000; Plonsky and Gass, 2011; Sato and Loewen, 2019). In contrast, low-level evidence holds little priority in deciding whether an intervention is effective for learners (see Plonsky and Gass, 2011, for review). Power and precision of treatment effect estimates have been gradually accepted (Oswald and Plonsky, 2010) and, more recently, required in L2 teaching research (Plonsky and Oswald, 2014; Marsden et al., 2018a, b).

**TABLE 1:** Levels of evidence for practical interventions.

**Research question: Does this intervention help?**	
Level 1a:	Systematic review with homogeneity of randomized controlled trials
Level 1b:	Individual randomized controlled trials
Level 2a:	Systematic review with homogeneity of cohort studies
Level 2b:	Individual cohort study including low-quality randomized controlled trials
Level 3a:	Systematic review with homogeneity of case–control studies
Level 3b:	Individual case–control study
Level 4:	Case series and poor-quality cohort and case–control studies
Level 5:	Expert opinion without explicit critical appraisal

*The guidelines are adapted from the therapy/prevention, etiology/harm column of Oxford Centre for Evidence-Based Medicine (2009). In this criterion, homogeneity refers to being free of worrisome degrees of results between individual studies, and studies displaying worrisome heterogeneity are tagged with a minus at the end of their designated level.*

There are two types of benchmarks for interpreting the magnitude of treatment effects in L2 teaching research. First, an L2-specific benchmark provides information on the general magnitude of treatment effects, as it is developed through the synthesis of whole domains of L2 instruction (Plonsky and Oswald, 2014). Second, treatment-specific benchmarks are based on specific domains of L2 instruction that have been separately synthesized, such as grammar teaching (Norris and Ortega, 2000), interaction (Plonsky and Gass, 2011), and extensive reading (Nakanishi, 2015; Jeon and Day, 2016). As these meta-analyses indicate that the effects of L2 teaching vary according to its approaches, treatment-specific benchmarks can be interpreted as the intrinsic effects of individual L2 instruction domains.

It is essential to refer to treatment-specific benchmarks when considering individual learners’ differences. Evidence-based practice requires empirical data on what works for whom (Mitchell, 2000; Chambless and Ollendick, 2001; Pachler, 2003; APA Presidential Task Force on Evidence-Based Practice, 2006; Sato and Loewen, 2019). In meta-analysis, moderator variables are introduced to represent learner characteristics (e.g., proficiency, age, and gender), as well as teaching differences (e.g., purpose, approach, and time on task). For example, Jeon and Day (2016) and Nakanishi (2015) showed differences in the effects of extensive reading according to learner characteristics, focused skills, length of instruction, and the implementation format (see Table 2). This information is useful to predict what forms of extensive reading work for what kinds of learners. For example, the effect of extensive reading on reading comprehension is between *d* = 0.54 (Jeon and Day, 2016) and *d* = 0.63 (Nakanishi, 2015). In other words, meta-analysis of L2 teaching research has the potential to identify specific variables, settings, and samples prospectively to determine as yet unknown treatment effects (Oswald and Plonsky, 2010).

**TABLE 2:** Different effects of extensive reading by moderator variables.

Moderators		Jeon and Day (2016)	Nakanishi (2015)	
		Between	Between	Pre-post
Participants	1. Middle school	0.35	–0.05	0.27
	2. High school		0.57	0.61
	3. University	0.70	0.48	1.12
	4. Adults		0.67	1.48
Focus skills	1. Reading speed	0.83	0.98	0.61
	2. Comprehension	0.54	0.63	0.72
	3. Vocabulary	0.47	0.18	1.25
Length	1. One semester	0.51	0.36	0.89
	2. Two semesters	0.59	0.52	0.74
	3. Over a year	0.60		1.92
Extensive	1. Exclusive activity	0.24		
reading format	2. Part of course	0.47		
	3. Part of curriculum	0.91		
	4. Extracurricular	0.67		

*Jeon and Day, 2016 categorized participants’ ages as adolescent (middle and high school level) and adults (university level and above).*

However, Seidler et al. (2019) criticized the retrospective nature of traditional meta-analysis because researchers’ knowledge of individual study results would influence the study selection process. Inconsistencies across individual studies in measurement methods also make the integration of data difficult. To solve these issues, they claimed the advantage of prospective meta-analyses, in which “studies are included prospectively, meaning before any individual study results related to the [prospective meta-analysis’] research question are known” (p. 1). This methodology is applied to a high priority research question only when previous evidence is limited, and new studies are expected to be conducted in the future. For example, evidence regarding the treatment effect of extensive reading is limited because of a lack of an appropriate comparison between treatment and control groups (Nakanishi, 2015). Although extensive reading has been accepted as part of L2 reading instruction because its statistical significance has been consistently reproduced, its possible effects in non-randomized controlled trials in prior studies have not been accurately analyzed (McLean and Rouault, 2017). This perspective will be a new research question such as how accurately the treatment effect of extensive reading can be assessed when using a study design that approximates randomized controlled trials. After defining a research question that has not been analyzed in primary studies, a systematic literature research, a synthesis of evidence, and an interpretation and reporting of results are conducted similar to the methods used in traditional systematic reviews. During this process, planned and ongoing studies eligible for inclusion are continuously added into the meta-analysis until the results can answer the research question (Pogue and Yusuf, 1998). For a more detailed explanation of and options for prospective meta-analyses, see Watt and Kennedy (2017) and Seidler et al. (2019).

In relation to the present study, one of the most critical problems with observational non-randomized data for the comparison of groups is selection bias or biased assignments of participants to treatment and control groups (Reichardt, 2009). This non-ignorable, non-randomized treatment assignment is likely to cause initial differences between the two groups in the assessment of treatment effects (Rubin, 1974). In the between-group design, therefore, we must confirm that selection bias in non-randomized data is reasonably ignorable to provide evidence that potential differences in outcome measures were not caused by selection differences extant before the treatment (e.g., Rubin, 1974; Rosenbaum and Rubin, 1983; Imai et al., 2008). Referring to descriptive statistics before adjusting outcome measures using any confounding variables may cause bias in the results of meta-analyses. For example, if control groups had higher L2 reading proficiency than treatment groups at the beginning of extensive reading, the differences between the two groups at the time of outcome measurements should be underestimated. Although some extensive reading research claimed between-group equivalence before the treatment (e.g., Beglar et al., 2012; Robb and Kano, 2013; Suk, 2017), the two meta-analyses on the topic (Nakanishi, 2015; Jeon and Day, 2016) did not examine how the primary studies attempted to reduce selection bias in between-group comparisons. Therefore, new studies that address possible selection bias are expected to emerge in the framework of a prospective meta-analysis.

### Propensity Score Analysis for Extensive Reading Research

Extensive reading is widely recognized as an effective approach to teaching reading in English as a foreign/second language (EFL/ESL) pedagogy. According to a systematic review (Day, 2015), the core principle of extensive reading is that L2 learners choose what they want to read and read as much as possible for pleasure, information, and general understanding. As criticized by Nakanishi (2015), there is no definition of extensive reading in terms of the number of books and words L2 learners read during the treatment. A variety of extensive reading formats have also been implemented according to teaching environments. For example, extensive reading is employed as an independent reading course, a part of reading course, a part of the curriculum, and an extracurricular activity (Nation and Waring, 2019). The most frequently used practice is supervised extensive reading, in which teachers help L2 learners choose reading materials and respond to their questions about the storyline, word and phrase meanings, and grammatical structures (Day, 2015). Jeon and Day’s (2016) meta-analysis showed that each extensive reading format contributed to improving L2 learners’ reading comprehension, fluency, and vocabulary knowledge except when it was implemented as an independent reading course.

Within the framework of evidence-based practice, however, empirical results from past extensive reading research have not been informative for theory development or pedagogical decision-making. Deficits in the assessment of treatment effects in this field have resulted in research bias and waste. L2 teaching research considers covariates possibly affecting treatment effect estimation using analysis of (co)variance and multiple regression analysis (see discussion in Plonsky and Gass, 2011). However, adjustment by means of these linear models constrains the number of confounding variables that can be controlled for because the inclusion of too many covariates in the models will make it difficult to estimate the treatment effect (e.g., Imai et al., 2008; Guo and Fraser, 2015). Instead, the current study applies a propensity score to adjust for variables that may confound the treatment effect estimation of extensive reading.

Propensity score matching – a method that has recently been adopted in medical, psychological, and educational research (Guo and Fraser, 2015; Leite, 2017), but not in L2 teaching research – is a statistical approach for reducing selection bias in treatment effect estimation by approximating complete randomized controlled trials (King and Nielsen, 2019). By definition, the treatment effect is the difference in the potential outcomes between individuals who are assigned to a treatment group and the same individuals who are assigned to a control group. However, this cannot be directly observed (Rubin, 1974). To solve this problem, Rosenbaum and Rubin (1983) developed the propensity score, or “the conditional probability of assignment to a particular treatment given a vector of observed covariates” (p. 41). This method is applied to balance the distribution of confounding variables between treatment and control groups by matching only those who have similar propensity scores.

Using the propensity score method, the average treatment effect (ATE; e.g., Imai et al., 2008) can be estimated as the effect of extensive reading on all treated and control individuals, similar to establishing the standardized mean differences between two groups. Schafer and Kang (2008) described the nature of the ATE as the average difference in potential outcomes between the groups in the following scenario: All participants are assigned to a treatment group, and then, they are assigned to a control group. Furthermore, by excluding students from a control group whose propensity score cannot be matched, the average effect on only those students who participated in the treatment can be estimated (Ho et al., 2011). This average treatment effect on the treated individuals (ATTs) is also important to consider in treatment effect assessment for pedagogical decision-making.

Learners’ initial L2 reading proficiency, L2 vocabulary size, and academic performance can be regarded as the confounding variables that cause selection bias in research on extensive reading. Since Jeon and Yamashita’s (2014) meta-analysis revealed that variances of L2 learners’ reading comprehension can be largely explained by cognitive aspects of reading, students with higher L2 reading proficiency and larger vocabulary size at the beginning of extensive reading should gain higher scores on the outcome measures. Reciprocal causation, where the amount of L2 reading increases as a result of motivation for engagement in extensive reading (Yamashita, 2004, 2007), should also be considered. When an extensive reading program is implemented as part of a course curriculum, students will be more dedicated to extensive reading in order to get higher grades and, accordingly, more likely to be proficient in L2 reading. Moreover, students will not only engage in extensive reading but also learn to read in L2 through other learning modes, such as vocabulary and grammar exercises in the classroom. Therefore, the outcome measures should reflect the treatment effects of classroom activities in addition to those of extensive reading. These covariate effects must be reduced to evaluate the treatment effect of extensive reading on L2 reading development accurately.

Reporting treatment effects of extensive reading, adjusted by propensity score methods, will be a key element of the protocol of a prospective meta-analysis. To mitigate the methodological deficits of extensive reading research designs (Nakanishi, 2015; McLean and Rouault, 2017), new studies applying propensity score methods similar to the current study are expected to emerge. Following a guide to prospective meta-analyses (Pogue and Yusuf, 1998; Watt and Kennedy, 2017; Seidler et al., 2019), the present study attempted to harmonize the design, implementation, and outcome collection of the planned studies. In Study 1, a meta-analysis was conducted to assess the selection bias in existing research on extensive reading and to estimate the expected effect size of extensive reading practice. In Study 2, a planned study using propensity score methods was integrated with the meta-analysis results. This methodology is a nested prospective meta-analysis, which integrates prospective evidence from planned study results into existing retrospective meta-analyses (Seidler et al., 2019).

## Study 1

### Method

#### Study Retrieval

Two large-scale meta-analyses on extensive reading (Nakanishi, 2015; Jeon and Day, 2016) were used to obtain synthesized effect sizes. Nakanishi (2015) included 34 studies using three keywords: *extensive reading*, *pleasure reading*, and *graded readers*. Jeon and Day (2016) updated this database in terms of the self-selected reading principle of extensive reading, and six studies were excluded because they offered obligatory assigned reading. In their meta-analysis, 21 studies from 1980 through 2014 were newly added.

In the present study, we conducted a search for the latest studies, written in English and published from April 2014 to April 2019. Five databases (Education Resources Information Center, Google Scholar, Linguistics and Language Behavior Abstracts, PsycINFO, and Web of Science) were electronically searched to locate relevant studies using the same keywords as Nakanishi (2015). After periodicals had been searched, full texts of book chapters, monographs, and relevant reports were also searched by citation chasing. This literature search found 47 studies published in 15 international peer-reviewed journals such as *Reading Research Quarterly*, *Studies in Second Language Acquisition*, *TESOL Quarterly*, and *Reading in a Foreign Language*. These studies were examined to determine whether they included information necessary for the present meta-analysis.

#### Criteria for Inclusion and Coding

The purpose of the inclusion criteria was to examine selection bias and to recalculate expected effect sizes to represent the present teaching environment. In Study 2, university students receiving English instruction were engaged in extensive reading for one semester as part of the curriculum, to improve their reading comprehension abilities. Their initial L2 reading proficiency was low [A1 level of the Common European Framework of Reference for Languages (CEFR)] as measured by a standardized reading test, TOEIC Bridge (Educational Testing Service, 2007). To select identified studies for the meta-analysis that were similar in terms of teaching and learner characteristics, the inclusion criteria were defined as follows:

All classification was duplicated in accordance with Nakanishi (2015) and Jeon and Day (2016). The existing 49 studies and the 47 newly collected studies were independently coded as below by two L2 reading researchers, with an intercoder agreement ratio of 92%. Any disagreements were resolved by reexamining the primary studies. Nineteen of the existing studies and three of the newly collected studies met the inclusion criteria (the primary studies included in the Present Meta-Analysis are presented in Supplementary Data Sheet 1). Statistical information to be analyzed was recorded by the author and checked by the other coder.

The primary studies included in the meta-analysis operationalized their extensive reading practice according to their teaching environment. For example, Suk (2017) implemented a 15-week semester extensive reading, in which Korean EFL students received 70 min of class time for intensive reading instruction that was similar to that received by the control group and the remaining 30 min for extensive reading activities. Some activities, such as scaffolded silent reading and writing a short book report, were incorporated to facilitate their reading during the class. These instructional procedures were similar to the present study and other primary studies (e.g., Al-Homoud and Schmitt, 2009; Nakanishi and Ueda, 2011; Beglar et al., 2012; Shih, 2015). Although some primary studies systematically promoted out-of-class extensive reading (e.g., Robb and Kano, 2013; Huffman, 2014; McLean and Rouault, 2017), we did not require our students to read outside class time because they were not independent learners.

#### Meta-Analysis

Standardized mean differences for between-group comparisons of outcome measures were calculated as an effect size of *d*. A random-effect model was applied to synthesize the effect sizes because the treatment effect of extensive reading differed according to various moderators (Nakanishi, 2015; Jeon and Day, 2016). Since four studies conducted multiple experiments using different samples (Sims, 1996; Mason and Krashen, 1997; Lee, 2007; Robb and Kano, 2013), data from each study were included in the meta-analysis separately, resulting in the resynthesis of 33 datasets from 22 primary studies, which included 6,806 participants (treatment, *n* = 3,343; control, *n* = 3,462).

Further meta-analysis explored the variance of standardized mean differences in pretests between treatment and control groups. A significant difference at the time of the pretests indicates selection bias related to inherent differences among participants. Eleven datasets from four primary studies did not include information on the descriptive statistics for the pretests; therefore, 22 datasets were submitted to meta-analysis (*N* = 1,998; treatment, *n* = 1,000; control, *n* = 998). For the moderator analysis, studies in which control groups had higher/lower L2 reading proficiency than treatment groups were labeled as “control” and “treated,” respectively, in cases where the 95% confidence intervals (CIs) of *d* did not include zero. Studies where the 95% *CIs* of *d* included zero were classified as “equivalent,” indicating that they used statistically equivalent groups for comparisons. Studies that did not include any information about pretest were categorized as “unspecified.” For the calculation of *d*, the means of control groups were subtracted from the means of treatment groups. The meta-analyses were executed with the metafor package for R (Viechtbauer, 2010)^1^.

**Table:** 

Criteria for inclusion – Studies that target EFL and ESL learners in high school, university, or educational institutions for adults and include their L2 proficiency information. – Studies that report a specific length of instruction. – Studies that use tests to measure learners’ reading comprehension abilities. – Studies that implement extensive reading as part of the curriculum. – Studies that report the numerical results obtained from between-group comparisons. – (Prospectively, studies that apply propensity score methods to estimate the treatment effect of extensive reading.)	Coding of study reports – Learner characteristics: EFL/ESL settings, school, and L2 reading proficiency self-labeled by each primary study^1^ (terms such as *beginner* and *novel* were categorized as lower proficiency; terms such as *intermediate* and *advanced* were categorized as higher proficiency). – Length of instruction: one semester, two semesters, and over a year (cf. *short, medium*, and *long*, Jeon and Day, 2016). – Tests used: a reading comprehension test and others. – Ways to implement extensive reading: an independent course, a part of a reading course, a part of a curriculum, an extracurricular activity, and others. – Research design: between-group comparison and others.

*^1^How to define participants’ proficiency and integrate the outcomes of participants defined by different measurements levels is a major challenge for meta-analysis of extensive reading (Nakanishi, 2015). Future research needs to use common measurements, and researchers should define participants’ proficiency levels using a common scale such as CEFR at least.*

### Results and Discussion

**FIGURE 1:**
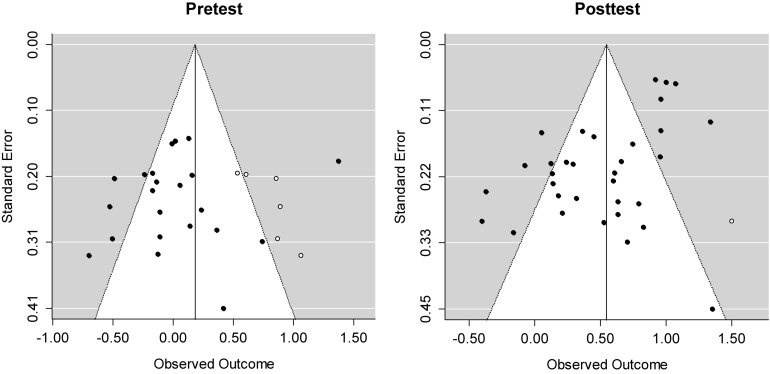
Funnel plots after applying a trim-and-fill method to reduce the effects of the existing publication bias. Standard errors on the *y*-axis indicate the precision of each study; the largest *N*-size studies have the smallest standard error. Effect sizes *d* for each study are plotted on the *x*-axis. Diagonal lines show the expected 95% confidence intervals around the summary estimate. White dots indicate the missing studies estimated by the trim-and-fill method.

Publication bias in the meta-analysis was assessed and found by a trim-and-fill method to estimate the number of missing studies because the number of published and unpublished studies was unequal (published = 18, unpublished = 3). Biased meta-analysis results lead to undesirable decisions about the treatment effect (e.g., Lipsey and Wilson, 1993; Plonsky and Oswald, 2014; Seidler et al., 2019). For the treatment effects (i.e., posttests), one missing study was added to adjust the underestimated effect size from 0.52 to 0.55. In the same way, six missing studies for the pretest data were added to recover the underestimated effect size from 0.02 to 0.18. Figure 1 shows that these adjustments resulted in symmetrical funnel plots.

The meta-analysis results showed a large variance in standardized mean differences between treatment and control groups at the time of pretests: *Min* = −0.71, 1-quantile = −0.19, *Mdn* = −0.06, 3-quantile = 0.18, *Max* = 1.38. The variance was positively skewed (skewness = 1.05), indicating that the primary studies were more likely to use control groups with higher L2 reading proficiency than the treatment groups before treatment. The moderator analysis results showed that the treatment effects of extensive reading differed according to the selection bias (Table 3). As expected, studies that used control groups whose initial L2 reading proficiency was higher than that treatment groups produced the lowest treatment effect [*d* = −0.24, 95% CI (−0.53, 0.05)]. Studies using treatment groups whose initial L2 reading proficiency was higher than control groups obtained higher treatment effects than the other two categories [*d* = 0.57, 95% CI (0.26, 0.87)]. Looking at the studies using the equivalent groups [*d* = 0.37, 95% CI (0.24, 0.50)], it is highly possible that selection bias caused under- or overestimations of the treatment effect of extensive reading. Note that the studies with no information about pretests greatly overestimated the treatment effect [*d* = 0.94, 95% CI (0.82, 1.05)].

**TABLE 3:** Results of the meta-analysis for the treatment effects of extensive reading.

		**Participants (*n*)**		**Effect sizes**		
**Moderators**	***k***	**Treatment**	**Control**	***d***	**95% CI**	***SE***
**Proficiency**						
Higher	18	2, 695	2, 797	0.71	(0.56, 0.86)	0.08
Lower	15	648	666	0.30	(0.12, 0.49)	0.09
**Instruction length**						
One semester	9	368	321	0.25	(0.04, 0.47)	0.11
Two semesters	16	733	776	0.45	(0.30, 0.60)	0.08
Over a year	8	2, 242	2, 366	0.92	(0.74, 1.09)	0.09
**Selection bias**						
Control	4	141	114	–0.24	(−0.53, 0.05)	0.15
Equivalent	15	691	724	0.37	(0.24, 0.50)	0.07
Treated	3	132	123	0.57	(0.26, 0.87)	0.16
Unspecified	11	2, 379	2, 501	0.94	(0.82, 1.05)	0.06
**Overall**	33	3, 343	3, 463	0.55	(0.39, 0.70)	0.08

**k*, number of studies, CI, confidence interval, SE, standard error.*

These findings suggest that the previous meta-analyses overestimated the treatment effect of extensive reading on L2 reading comprehension skills (see Table 2, Focus skills, Comprehension: *d* = 0.54 in Jeon and Day, 2016; *d* = 0.63 in Nakanishi, 2015). Accordingly, the treatment effects of extensive reading accumulated so far are minimally informative for theories and pedagogical decision-making within the framework of evidence-based practice. Although the use of between-group designs has been recommended due to an inflation effect caused by pre–posttest designs in L2 teaching research (e.g., Plonsky and Oswald, 2014; Mackey and Gass, 2015; Sato and Loewen, 2019), the findings of the present study further indicate the importance of ensuring between-group equivalence by controlling participant factors that may affect outcome measures.

Before considering selection bias, Table 3 showed that the overall effect size was 0.55 [95% CI (0.39, 0.70)]. This treatment effect was expected to decrease when targeting beginner-level students [*d* = 0.30, 95% CI (0.12, 0.49)] and implementing one-semester extensive reading [*d* = 0.25, 95% CI (0.04, 0.47)]. In Study 2, we conducted a study using propensity score methods to compare the treatment effects with the benchmarks established in Study 1. The results of Study 2 were not known before defining the present inclusion criteria, and it was fully eligible for inclusion in the meta-analysis. It is the key feature of a prospective meta-analysis that studies are identified as eligible for inclusion before those results are known (Pogue and Yusuf, 1998; Seidler et al., 2019). By including such planned studies that adopt propensity score methods to estimate the treatment effect of extensive reading, a prospective meta-analysis can largely eliminate biased effect sizes.

## Study 2

### Method

#### Participants

We used a non-randomized controlled trial that included five intact EFL classes, and 224 Japanese EFL learners participated in Study 2 (age = 18–19 years). Two classes were assigned to a control group (*n* = 115; female = 77, male = 38), where the general aim of the course was to improve English speaking and writing skills. The other two classes – the treatment group – engaged in extensive reading (*n* = 109; female = 67, male = 42). Participants were first-year undergraduates majoring in nursing (treatment, *n* = 43; control, *n* = 46), physiotherapy (treatment, *n* = 66; control, *n* = 44), and child education (control, *n* = 25). By the beginning of this study, they had received 6 years of English instruction as part of their formal education in Japanese secondary schools and had not experienced any extensive reading activities. Before the treatment, informed consent was obtained, and the participants were notified of how the personal data collected would be used.

The participants were obligatorily enrolled in a weekly 90-min basic English skills course at their university. Their English reading proficiency was assessed using a 50-item standardized reading test, TOEIC Bridge (score range = 10–90; Educational Testing Service, 2007) before the treatment (at the beginning of the academic year). Their dichotomously marked reading test score showed that they were at the A1 level of the CEFR [*M* = 42.00, 95% CI (39.67, 44.33), SD = 17.70, Cronbach’s α = 0.83], indicating that the participants were not independent readers (Educational Testing Service, 2019).

#### Materials

The reading texts offered for extensive reading were derived from the short reading passages compiled by the Eiken Foundation of Japan. Although books such as graded readers are more appropriate for extensive reading, the length of these books may intimidate A1-level L2 readers. Nuttall (2005) recommended the use of short, appealing, varied, and easy passages for elementary readers. Accordingly, three positive reasons for using the EIKEN reading passages were as follows: (a) the reading texts were simplified in terms of word frequency and syntactic complexity, (b) the EIKEN grades were associated with the CEFR level, and (c) the text characteristics were synchronized with the Course of Study of English in Japan (see Table 4). Twenty-six different texts were prepared for seven grades, resulting in a total of 182 reading passages. Text genres included narrative, scientific expository, essay, and everyday language, such as emails, notices, and advertisements.

**TABLE 4:** EIKEN grades and their Common European Framework of Reference for Languages (CEFR) level with text variables.

**EIKEN grade**	**CEFR level**	**EIKEN benchmark**	**Mean standard words**		**Flesch–Kincaid grade level**	
			***M***	***SD***	***M***	***SD***
Grade 2	B1	High school/graduates	367.45	12.56	9.25	1.02
Grade Pre-2	A2	High school/intermediates	307.38	8.81	8.31	0.90
Grade 3	A1	Junior high school/graduates	258.25	12.30	6.76	1.29
Grade 4	A1	Junior high school/intermediates	155.60	5.78	4.23	0.99

*Each grade has 26 different kinds of passages.*

Two versions of standardized reading comprehension tests (Educational Testing Service, 2007) were used to measure participants’ L2 reading proficiency at the beginning and end of the extensive reading. They consisted of 30 multiple-choice comprehension questions with 20 passages from various genres such as articles, emails, notices, forms, reports, and advertisements. To avoid testing and instrumentation effects (Reichardt, 2009), one treatment and one control group took the two tests in normal order (Test A for the pretest; Test B for the posttest), while the other two groups took them in reverse order (Test B for the pretest; Test A for the posttest). The reliability coefficients of the pretest (Cronbach’s α = 0.83) and posttest (Cronbach’s α = 0.89) were high.

The 1,000- to 5,000-word level of a standardized vocabulary test (Koizumi and Mochizuki, 2011) was used to measure participants’ L2 vocabulary size before the treatment. This test – 125 multiple-choice questions – was developed to assess the written receptive vocabulary knowledge of Japanese EFL learners. In each question, participants were given a Japanese word and instructed to select the most appropriate English translation from three options. The reliability coefficient was high (Cronbach’s α = 0.95).

Participants’ academic performance in a regular English class was evaluated using the average scores of two end-of-term tests prior to the treatment. The tests consisted of integrated reading-to-writing task performance (50%), independent listening skills (15%), independent reading skills (15%), and spoken interaction (20%).

#### Procedure

Course work for the treatment group was broadly divided into two activities. For about 60 min in class, the treatment group relearned, through task-based language learning, grammatical and vocabulary items that had been introduced in junior and senior high schools. For the remaining 30 min, they engaged in the extensive reading at their own pace.

In the extensive reading segment, the participants were initially instructed to read EIKEN Grade 3 reading texts. After reading three texts from each grade, the participants were free to move on to higher levels; however, they were advised to read texts at lower levels if they had difficulty comprehending content. During class, they chose a reading text and engaged in sustained silent reading. Every time they finished reading a text, they briefly shared their thoughts about the contents by writing a short book report, then returned the text and took a new one for additional reading. To confirm that students had read the texts and to motivate extensive reading, a teacher monitored reading progress and answering any comprehension questions, writing brief comments after each class. Following Beglar et al. (2012), the total amount of reading by all participants was calculated using standard words comprising six characters as a nominal word.

#### Data Analysis

The main steps of propensity score analysis include propensity score estimation, matching and covariate balance evaluation, and treatment effect estimation (Leite, 2017). The included covariates should be true confounders that are measured before treatment assignment or are stable over time (e.g., Rosenbaum and Rubin, 1983; Imai et al., 2008; Ho et al., 2011). For propensity score estimation, this study considered as many variables as possible that could potentially determine students’ participation in the treatment group. We included the following five covariates obtained before treatment: (a) initial L2 reading proficiency, (b) L2 vocabulary size, (c) academic performance, (d) gender, and (e) major in school. Although both gender and academic major were assumed not to be predictors of outcome, these were true confounders affecting the probability of treatment assignments in a non-randomized study. In other words, because the participants’ gender and school faculty were not randomized when we assigned them into either treatment or control groups, both covariates were included in the analysis. Therefore, these five covariates were submitted to a stepwise logistic regression model, and propensity scores were estimated.

Propensity score matching was conducted for group participants with similar propensity scores. Since there are different matching methods, it is necessary to choose a method that shows the best balance of covariates and propensity scores. We employed and compared six different matching methods: nearest neighborhood matching without replacement, nearest neighborhood matching with replacement, genetic matching without replacement, genetic matching with replacement, optimal nearest neighborhood matching, and unconstrained full matching. For details about each matching method, see, for example, Leite (2017).

Next, both ATE and ATT were estimated. In this study, the ATE was the difference between the expected posttest values of all the participants in the treatment and control groups. The ATT was the difference between the expected posttest values of the participants in the treatment group only. The purpose of this study was to evaluate whether extensive reading was beneficial for those learners who were assigned to the treatment group (i.e., ATT) as well as whether, on average, extensive reading was beneficial for all the participants (i.e., ATE). The matching and treatment effect estimation were conducted with the MatchIt (Ho et al., 2011) and Matching (Sekhon, 2011) packages for R.

Finally, a sensitivity analysis was conducted to reveal how strongly the unidentified covariates would affect the significance test of the treatment effect. Evaluating sensitivity to the unidentified covariates is important because propensity score methods only reduce selection bias caused by observed covariates (Liu et al., 2013). The rbound package for R (Keele, 2014) was used for Rosenbaum’s (2002) method to calculate *p*-values that showed how sensitive the results of treatment effect estimations were to the unidentified covariates.

### Results and Discussion

Table 5 displays the descriptive statistics of the pre- and posttest results for the treatment and control groups. The treatment group read an estimated 25,000 standard words on average (*Min* = 11,630, 1-quantile = 18,235, *Mdn* = 23,505, 3-quantile = 26,865, *Max* = 42,985). A two-tailed *t*-test showed no significant difference in the posttest score between the two groups before applying the propensity score matching, *t*(222) = 1.64, *p* = 0.103, *d* = 0.22. This result can be attributed to the selection bias in this study because the control group was always better than the treatment group at initial L2 reading proficiency, L2 vocabulary size, and academic performance. These confounding variables affecting the treatment effect estimation complicated pedagogical interpretations, even though the pre–postgain score of the reading test was higher in the treatment group (*M* = 4.71) than in the control group (*M* = 0.93). These results suggest the necessity to control covariates by propensity score analysis.

**TABLE 5:** Descriptive statistics for reading tests, L2 vocabulary size, and academic performance.

	**Treatment (*n* = 109)**			**Control (*n* = 115)**		
**Measures**	***M***	**95% CI**	***SD***	***M***	**95% CI**	***SD***
Pretest	7.19	(6.33, 8.06)	4.56	10.98	(10.28, 11.68)	3.67
Posttest	11.90	(10.98, 12.81)	4.97	11.91	(11.04, 12.79)	4.74
L2 vocabulary size	2704.45	(2562.82, 2846.07)	745.95	3311.38	(3193.46, 3429.31)	638.36
Academic performance	72.23	(70.39, 74.07)	9.69	79.80	(77.17, 82.43)	14.25

For propensity score estimation, logistic regression results showed that initial L2 reading proficiency (*B* = −0.198, SE = 0.042, *p* < 0.001), L2 vocabulary size (*B* = −0.001, SE = 0.000, *p* < 0.001), academic performance (*B* = −0.084, SE = 0.016, *p* < 0.001), and academic major (*B* = −1.973, SE = 0.348, *p* < 0.001) explained 46% of variance of the treatment assignment probability. Participants’ gender was not a strong predictor of the treatment assignment (*B* = −0.402, SE = 0.210, *p* = 0.056). The rank discrimination index showed that prediction by this logistic model was good [c-index = 0.89, 95% CI (0.85, 0.93)]. Thus, these four covariates were used in propensity score matching.

**FIGURE 2:**
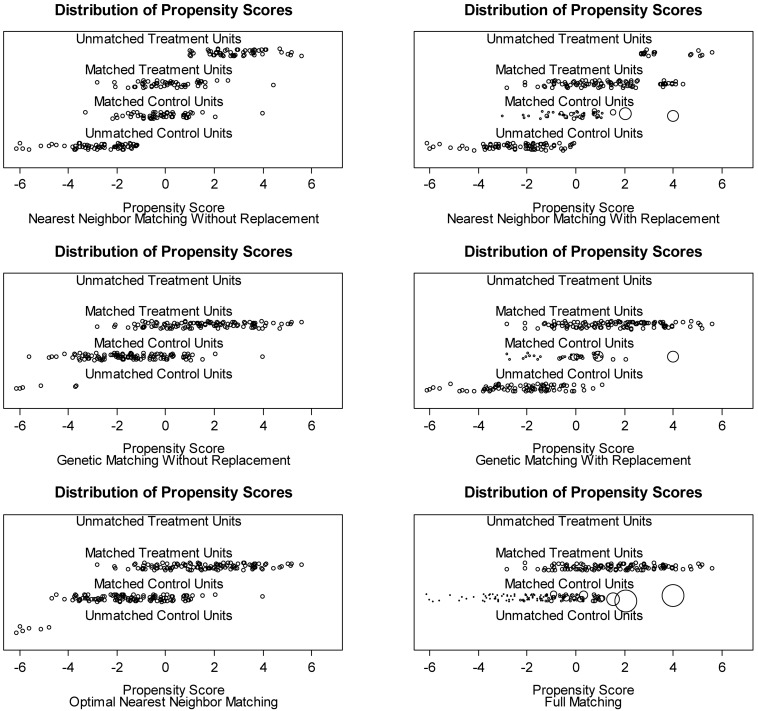
Jitterplots displaying the distribution of propensity scores after six different matchings. Circle sizes indicate the assigned weights for group comparison regarding the treatment effect.

To select the best matching procedure, this study explored change in the absolute standardized mean differences of the propensity scores between before and after matchings. According to Leite (2017), when the absolute values of propensity scores are <0.10, covariate balances are strict, and when the absolute values are <0.25, covariate balances are lenient. Table 6 shows that nearest neighborhood matching with replacement (0.03) and full matching (0.05) satisfied the criterion for “strict.” Nearest neighborhood matching without replacement (0.21) satisfied the criterion for “lenient.” Figure 2 presents the propensity score distribution after six matching procedures, demonstrating whether there was sufficient propensity score overlap between the treatment and control groups. For example, nearest neighborhood matching with replacement, nearest neighborhood matching without replacement, and full matching all showed high overlap of the propensity scores for the matched treatment and control groups. By contrast, the other three matching procedures did not produce similarities between the matched groups. The treatment effect estimation was conducted based on these three matching procedures.

**TABLE 6:** Differences in means of confounding variables by propensity score matching.

**Matching methods**	**Treatment**	**Control**	**Standardized mean difference**
**Before matching (Treatment: *n* = 109, control: *n* = 115)**			
Propensity score	1.58	–1.61	1.73
Initial L2 reading proficiency	7.19	10.98	1.03
L2 vocabulary size	2704.45	3311.38	0.81
Academic performance	72.23	79.80	0.78
Academic major	1.61	1.90	0.77
**Nearest neighborhood matching without replacement (Treatment: *n* = 54, control: *n* = 54)**			
Propensity score	0.17	–0.22	0.21
Initial L2 reading proficiency	9.43	9.83	0.09
L2 vocabulary size	3130.69	3138.89	0.01
Academic performance	73.67	75.89	0.23
Academic major	1.72	1.78	0.11
**Nearest neighborhood matching with replacement (Treatment: *n* = 91, control: *n* = 41)**			
Propensity score	1.17	1.11	0.03
Initial L2 reading proficiency	7.67	7.38	0.06
L2 vocabulary size	2817.62	3327.00	0.68
Academic performance	72.52	71.27	0.13
Academic major	1.67	1.41	0.54
**Genetic matching without replacement (Treatment: *n* = 109, control: *n* = 109)**			
Propensity score	1.58	–1.42	1.63
Initial L2 reading proficiency	7.19	11.47	0.94
L2 vocabulary size	2704.45	3297.82	0.80
Academic performance	72.23	79.22	0.72
Academic major	1.61	1.96	0.73
**Genetic matching with replacement (Treatment: *n* = 109, Control: *n* = 34)**			
Propensity score	1.58	1.05	0.26
Initial L2 reading proficiency	7.19	6.96	0.05
L2 vocabulary size	2704.45	2937.41	0.31
Academic performance	72.23	73.32	0.11
Academic major	1.61	1.68	0.15
**Optimal nearest neighborhood matching (Treatment: *n* = 109, Control: *n* = 109)**			
Propensity score	1.58	–1.39	1.61
Initial L2 reading proficiency	7.19	11.47	0.94
L2 vocabulary size	2704.45	3295.35	0.79
Academic performance	72.23	79.58	0.76
Academic major	1.61	1.94	0.67
**Full matching (Treatment: *n* = 109, Control: *n* = 115)**			
Propensity score	1.58	1.49	0.05
Initial L2 reading proficiency	7.19	6.63	0.12
L2 vocabulary size	2704.45	3399.81	0.93
Academic performance	72.23	67.78	0.46
Academic major	1.61	1.39	0.45

Tables 7, 8 summarize the ATEs and the ATTs of extensive reading on L2 reading improvement, estimated by the three matching procedures, respectively. Effect sizes were calculated based on the mean differences between the treatment and control groups and the pooled standard deviations of the posttest. In Table 7, with regard to the ATE estimation after three matchings, the treatment effect increased from 0.22 (i.e., the effect size *d* calculated before ensuring between-group equivalence) to 0.24–0.44. More importantly, as shown in Table 8, the ATT results showed that, when matched on all covariates, the treated students’ L2 reading proficiency improved significantly more than control students (*d* range = 0.32–0.40). These effect sizes were consistent with the results of the meta-analysis using the studies that ensured between-group equivalence (*d* = 0.37; see Table 3).

**TABLE 7:** The average treatment effects (ATEs) of the extensive reading for different matching methods.

**Matching methods**	**Treatment**		**Control**			
	***M***	***SD***	***M***	***SD***	**ATE**	***d***
Nearest neighborhood matching without replacement	12.47	2.86	9.66	2.81	2.81	0.34
Nearest neighborhood matching with replacement	11.94	3.07	8.96	3.14	2.98	0.44
Full matching	12.87	2.66	10.17	2.67	2.61	0.24

**TABLE 8:** The average treatment effects on the treated individuals (ATTs) of the extensive reading for different matching methods.

**Matching methods**	**Estimate**	***SE***	***t***	***p***	***d***
Nearest neighborhood matching without replacement	2.85	0.75	3.83	0.000	0.35
Nearest neighborhood matching with replacement	2.69	1.14	2.36	0.020	0.40
Full matching	3.64	0.82	4.47	0.000	0.32

Finally, the results of the sensitivity analysis are shown in Table 9. According to Rosenbaum (2002), the value of gamma is interpreted as odds ratios of different probabilities of treatment assignment. If this value is close to 1, the estimated treatment effect is sensitive to unidentified covariates. In particular, a change in the lower and higher bounds of *p*-values from significant to insignificant (or vice versa) indicates the exact value of gamma to be discussed. Although this analysis can be generalized for matching procedures beyond one-to-one matching, it is not as easily implemented by the existing statistical software (Keele, 2014). Therefore, note that the sensitivity analysis with one-to-one greedy matchings (i.e., the nearest neighborhood matchings with and without replacement) was conducted but not with full matching. The results showed that, in both matching procedures, the higher bound estimates changed from significant to insignificant when gamma was 1.8. It is difficult to conclude whether the effects of unidentified covariates are present because the Rosenbaum’s sensitivity analysis does not provide any objective criteria (e.g., Imai et al., 2008; Liu et al., 2013). However, the present results will be more robust against unidentified covariates if a large change in the odds ratio is needed by adding the covariates, theoretically affecting the treatment assignment of the extensive reading program.

**TABLE 9:** Results of the Rosenbaum’s sensitivity analysis for the Wilcoxon’s signed rank test.

**Gamma**	**Nearest neighborhood** **matching without** **replacement**		**Nearest neighborhood** **matching with** **replacement**	
	**Lower bound**	**Higher bound**	**Lower bound**	**Higher bound**
1.0	0.0008	0.0008	0.0009	0.0009
1.1	0.0003	0.0019	0.0003	0.0023
1.2	0.0001	0.0040	0.0001	0.0048
1.3	0.0000	0.0076	0.0000	0.0089
1.4	0.0000	0.0130	0.0000	0.0150
1.5	0.0000	0.0206	0.0000	0.0236
1.6	0.0000	0.0307	0.0000	0.0349
1.7	0.0000	0.0434	0.0000	0.0491
1.8	0.0000	0.0589	0.0000	0.0661
1.9	0.0000	0.0770	0.0000	0.0860
2.0	0.0000	0.0977	0.0000	0.1085

*Gamma values refer to odds ratios of differential assignment to treatment due to unidentified covariates. Lower and higher bounds mean the intervals of *p*-values based on the Wilcoxon’s signed rank statistics for the outcome difference between treatment and control groups (Rosenbaum, 2002).*

## General Discussion

The purpose of this study was to propose the method of treatment effect assessment toward the establishment of an evidence-based practice in extensive reading. In Study 1, the existing two meta-analysis studies were reassessed for selection bias associated with primary studies to determine their quantitative reproducibility with regard to the practical significance of extensive reading. When including only the studies that ensured between-group equivalence, the effect size expected for the present extensive reading study was 0.37 [95% CI (0.24, 0.50)], indicating that the previous meta-analyses overestimated treatment effect. In Study 2, this estimation was validated by applying propensity score methods. By reducing the selection bias, this study produced ATEs and ATTs consistent with the meta-analysis results. These findings show that new primary studies should be planned for inclusion into prospective meta-analyses.

Systematic reviews and meta-analyses of the best available research evidence have the potential to inform pedagogical decision-making for L2 teaching. The current study, however, revealed that the retrospective nature of previous meta-analyses included biased interpretations regarding the treatment effect of extensive reading. The results showed significant differences in the effect sizes between studies that ensured between-group equivalence and those that did not. As many researchers have indicated that primary studies on extensive reading include methodological problems (e.g., Al-Homoud and Schmitt, 2009; Beglar et al., 2012; McLean and Rouault, 2017; Suk, 2017), the current status of existing extensive reading research is that it introduces bias and waste. In addition to future research including detailed descriptive statistics and control groups, as recommended by Nakanishi (2015), primary studies must ensure between-group equivalence by random assignment (McLean and Rouault, 2017) and by embedding propensity score adjustment in the planned research.

The current study adopted propensity score methods appropriate for addressing treatment effect estimation of extensive reading. Propensity score matching was conducted to reduce selection bias associated with possible confounding variables. The list of observed pretreatment covariates included the factors affecting outcome measures, typically considered by previous studies on extensive reading (Yamashita, 2004, 2007, 2015; Day, 2015; Waring and McLean, 2015). By matching the propensity scores between the treatment and control groups, the target population of students was defined in order to generalize causal inference about the effects of extensive reading in L2 settings. The results of the ATEs and ATTs both validated the causal inference that students who participated in extensive reading improved their L2 reading comprehension skills more than students who did not participate in the program. Following the L2-specific benchmark for effect sizes (Plonsky and Oswald, 2014), the treatment effect of extensive reading was small when the focused skill of the one-semester program for EFL students was reading comprehension (ATEs, *d* = 0.24–0.44; ATTs, *d* = 0.32–0.40). This is consistent with the primary studies that ensured the between-group equivalence (e.g., McLean and Rouault, 2017; Suk, 2017). Although the interpretation is disputable that empirical research ends in failure when the reproduced effect size is significantly lower than the meta-analyzed effect size, at least some pedagogical decision-making is necessary about why interventions are ineffective.

The robust results for meta-analyses of treatment effects are essential to implement evidence-based practice in L2 pedagogy. With respect to extensive reading, Beglar et al. (2012) pointed out that past research reporting treatment effects depended on null hypothesis significance testing. Marsden et al. (2018a) also demonstrated that the extent of reproducibility of primary L2 teaching research depended on a narrative comparison of the findings and dichotomous judgment based on null hypothesis significance testing. The present study showed the importance of considering the degree to which treatment effect would be expected in L2 teaching, based on meta-analysis. In particular, moderator analysis was used to inform variability and predictability of treatment effects of extensive reading (see also Nakanishi, 2015; Jeon and Day, 2016). This treatment effect assessment provides research evidence to interpret to what extent particular L2 teaching formats work successfully and for whom. As suggested by Oswald and Plonsky (2010), effect sizes predicted *a priori* must be used as criteria for interpreting the outcomes of L2 teaching. Research-based evidence will help reject over- or underestimates of the treatment effects reported in literature (Oswald and Plonsky, 2010).

The current extensive reading research was integrated in the two retrospective meta-analyses as part of the nested prospective meta-analysis suggested by Seidler et al. (2019). Given that new studies meeting the inclusion criteria are included in prospective meta-analyses until generalizability of findings is achieved (Pogue and Yusuf, 1998), prospective study registration is necessary to complete the current prospective meta-analysis. This approach can be useful in L2 teaching research because Marsden et al. (2018b) suggested participation in the open science movement by introducing registered reports of primary research in this field. L2 teaching researchers should therefore be encouraged to submit the full method and analysis protocol of their studies prior to data collection. Moreover, prospective meta-analyses encourage the inclusion of studies by providing information regarding the defined research question and eligibility criteria (Seidler et al., 2019). For example, the prospective meta-analysis proposed in this study requires more ongoing studies that use propensity score methods for treatment effect estimation of extensive reading. L2 teaching researchers can now plan their primary studies for prospective integration into the meta-analysis.

The present study had a limited quantitative focus on evidence-based practice. Moderator analysis will improve language teaching expertise because it provides information about what teaching methods work for whom. For example, the present results showed that the treatment effects of extensive reading changed according to participants’ proficiency, focused skills, length of instruction, and implementation format (see also Nakanishi, 2015; Jeon and Day, 2016). However, a qualitative approach to decision-making on treatment effects is also necessary because sociocultural aspects, such as understanding the influence of individual and cultural differences on treatment (APA Presidential Task Force on Evidence-Based Practice, 2006), are often examined in qualitative studies, and these aspects should be examined as well in relation to extensive reading. Future studies should use a mixed-methods approach when examining the treatment effect of evidence-based practice in L2 pedagogy in conjunction with teacher cognition involved in pedagogical decision-making.

A statistical point that should be discussed is covariate selection. The pretreatment variables used as covariates in this study were mainly related to cognitive aspects in extensive reading. However, Yamashita (2004; 2007; 2015) highlighted the role of psychological aspects in L2 reading, such as reading attitude, motivation, and anxiety, affecting both participation in an extensive reading program and outcome measures. Hamada and Takaki (2019) also discussed the covariate effects of L2 reading anxiety on L2 reading proficiency. As the sensitivity analysis results implied that the assumption of ignorable treatment assignment (e.g., Rosenbaum and Rubin, 1983; Imai et al., 2008) was not fully applied in the current study, there is a need for further research that assesses all of the background variables relevant for treatment assignment. When selecting covariates in propensity score analysis, King and Nielsen (2019) recommend including (a) important covariates to cause selection bias, (b) information about how much imbalance caused by the covariates is left, and (c) a sample size still large enough after matching. Although the imbalance observed in the present study was adjusted by the propensity scores, the sample size for the treatment effect estimation consequently became smaller following the nearest neighborhood without and with replacement matchings (see Table 6). The thorough application of propensity score analysis is beyond the scope of this study; however, it will be necessary to replicate the results using the same research design.

In terms of implications for evidence-based practice in extensive reading in L2, the most essential contribution of this study is its attempt to advance the assessment theory of treatment effects for the integration of the best available research evidence into extensive reading activities in an intact class. Whereas Mitchell (2000) and Pachler (2003) critically discussed some difficulties in incorporating evidence-based practice in L2 teaching with educational policymaking, they suggested the applicability of research findings to classroom-based practice (see also Sato and Loewen, 2019). Among the many concerns regarding the implementation of evidence-based practice (see Pachler, 2003), it is important to synthesize and summarize existing research evidence (Chambless and Ollendick, 2001), assess the levels of evidence quality (Oxford Centre for Evidence-Based Medicine, 2009), and acquire the best available research evidence as expertise (APA Presidential Task Force on Evidence-Based Practice, 2006).

Plonsky and Oswald (2014) recommended reviewing L2 teaching research to consider using meta-analysis as a procedure for pedagogical decision-making. In the case of extensive reading, Nakanishi (2015) and Jeon and Day (2016) provided the list of aggregated primary research coded by a well-structured scheme. In the same way, various L2 teaching researchers have published a bibliography with coding information, ranging from specific L2 instruction to educational programs. This research trend helps when moving from retrospective to prospective meta-analyses. In working toward evidence-based practice in L2 pedagogy, it is necessary to accumulate better quality research evidence by including planned, well-designed, and registered research in meta-analyses. While aggregated evidence in L2 teaching has so far been assessed by systematic review through retrospective meta-analysis, prospective meta-analyses require registered reports adhering to previously defined eligibility criteria. The best available research evidence obtained from prospective meta-analyses can be applied to pedagogical decision-making in individual classrooms. To this end, treatment effect assessment will strongly contribute to advancing L2 teaching research toward evidence-based practice.

## Conclusion

This study focused on how to embed research evidence into classroom-based L2 teaching within the framework of evidence-based practice. The results showed that the effect sizes synthesized by moderator analysis could predict the treatment effects of L2 teaching for individual classrooms. The importance of research-based practice has been emphasized in foreign language education (Mitchell, 2000; Pachler, 2003; Sato and Loewen, 2019). To move toward evidence-based practice in L2 pedagogy, it is necessary to establish a virtuous cycle to (a) assess the levels of scientific evidence obtained from individual research, (b) acquire L2 teaching expertise from best available research evidence, and (c) apply it to other classrooms to provide further research evidence. This study suggests that planned and ongoing L2 teaching studies applying propensity score methods should be registered for inclusion into prospective meta-analyses. This methodological approach to treatment effect assessment helps reduce research bias and waste while also improving pedagogical decision-making based on efficient, adaptive, and collaborative use of educational data. The present findings provide strong support for this approach by demonstrating that the treatment effects of L2 teaching are reproducible when planning teaching procedures based on research evidence.

## Data Availability Statement

The datasets generated for this study are available in the IRIS digital repository (https://www.iris-database.org/iris/app/home/detail?id=york%3a937791&ref=search).

## Ethics Statement

The studies involving human participants were reviewed and approved by the Research Ethics Committee of the Faculty of Humanities and Social Sciences of the University of Tsukuba. The patients/participants provided their written informed consent to participate in this study.

## Author Contributions

The author confirms being the sole contributor of this work and has approved it for publication.

## Conflict of Interest

The authors declare that the research was conducted in the absence of any commercial or financial relationships that could be construed as a potential conflict of interest.

## Footnotes

1 All raw data and the R scripts used for the meta-analysis and propensity score analysis are available to readers in the IRIS digital repository (https://www.iris-database.org/iris/app/home/detail?id=york%3a937791&ref=search).
