# Supplementary material for: Using Meta-Analysis and Propensity Score Methods to Assess Treatment Effects Toward Evidence-Based Practice in Extensive Reading
Source: Front Psychol. 2020 Apr 22;11:617. doi: 10.3389/fpsyg.2020.00617 (PMC7188915; doi:10.3389/fpsyg.2020.00617)
Supplement: Supplementary file 1 [file Data_Sheet_1.docx]

Supplementary Material

*Primary Studies Included in the Present Meta-Analysis*

Al-Homoud, F., and Schmitt, N. (2009). Extensive reading in a challenging environment: A comparison of extensive reading and intensive reading approach in Saudi Arabia. Lang. Teach. Res. 13, 383–401. doi: 10.1177/1362168809341508

Beglar, D., Hunt, A., and Kite, Y. (2012). The effect of pleasure reading on Japanese university EFL learners’ reading rates. Lang. Learn. 62, 665–703. doi: 10.1111/j.1467-9922.2011.00651.x

Bell, T. (2001). Extensive reading: Speed and comprehension. Read. Matrix. 1(1), 1–13.

Burrows, L. (2012). The effects of extensive reading and reading strategies on reading self-efficacy. [dissertation]. [Tokyo (Japan)]: Temple University

Chen, C.-N., Chen, S.-C., Chen, S.-H. E., and Wey, S.-C. (2013). The effects of extensive reading via e-books on tertiary level EFL students’ reading attitude, reading comprehension, and vocabulary. Turk. Online J. Educ. T. 12, 303–312.

De Morgado, N. F. (2009). Extensive reading: Students’ performance and perception. Read. Matrix. 9, 31–43.

Huffman, J. (2014). Reading rate gains during a one-semester extensive reading course. Read. Foreign Lang. 26, 17–33.

Jeon, J. (2008). Extensive reading in a formal English reading class. Engl. Teach. 63, 49–83.

Lee, S.-Y. (2007). Revelations from three consecutive studies on extensive reading. RELC J. 38, 150–170. doi: 10.1177/0033688207079730

Lin, L.-F. (2010). Senior high school students’ reading comprehension of graded readers. J. Lang. Teach. Res. 1, 20–28.

Mason, B., and Krashen, S. (1997). Extensive reading in English as a foreign language. Syst. 25, 91–102. doi: 10.1016/S0346-251X(96)00063-2

McLean, S., and Rouault, G. (2017). The effectiveness and efficiency of extensive reading at developing reading rates. Syst. 70, 92–106. doi: 10.1016/j.system.2017.09.003

Nakanishi, T., and Ueda, A. (2011). Extensive reading and the effect of shadowing. Read. Foreign Lang. 23, 1–16.

Rezaee, A. A., and Nourzadeh, S. (2011). Does extensive reading improve EFL learners’ processing ability? Theory Pract. Lang. Stud. 1, 1167–1175. doi: 10.4304/tpls.1.9.1167-1175

Robb, T. N., and Kano, M. (2013). Effective extensive reading outside the classroom: A large-scale experiment. Read. Foreign Lang. 25, 234–247.

Robb, T. N., and Susser, B. (1989). Extensive reading vs. skills building in an EFL context. Read. Foreign Lang. 5, 239–251.

Shih, Y.-C. (2015). The impact of extensive reading on college business majors in Taiwan. Read. Matrix. 15, 220–233.

Sims, J. M. (1996). A comparative study of improvements in reading comprehension of skill-based instruction and extensive reading for pleasure with Taiwanese freshmen university students. [dissertation]. [Tallahassee (FI)]: Florida State University.

Smith, K. (2006). A comparison of “pure” extensive reading with intensive reading and extensive reading with supplementary activities. Int. J. Foreign Lang. Teach. 2, 12–15.

Suk, N. (2017). The effects of extensive reading on reading comprehension, reading rate, and vocabulary acquisition. Read. Res. Q. 52, 73–89. doi: 10.1002/rrq.152

Tanaka, H., and Stapleton, P. (2007). Increasing reading input in Japanese high school EFL classrooms: An empirical study exploring the efficacy of extensive reading. Read. Matrix. 7, 115–131.

Weitz, W. E. (2003). Sustained silent reading with non-native speakers of English: Its impact on reading comprehension, reading attitude, and language acquisition. [dissertation]. [Los Angeles (CA)]: University of Southern California.
