# Supplementary material for: Using Meta-Analysis and Propensity Score Methods to Assess Treatment Effects Toward Evidence-Based Practice in Extensive Reading
Source: Front Psychol. 2020 Apr 22;11:617. doi: 10.3389/fpsyg.2020.00617 (PMC7188915; doi:10.3389/fpsyg.2020.00617)
Supplement: Supplementary file 2 [file Data_Sheet_2.docx]

Supplementary Material

|  |  |
| --- | --- |

**Figure 1.** Funnel plots after applying a trim-and-fill method to reduce the effects of the existing publication bias. Standard errors on the y-axis indicate the precision of each study; the largest *N*-size studies have the smallest standard error. Effect sizes *d* for each study are plotted on the x-axis. Diagonal lines show the expected 95% confidence intervals around the summary estimate. White dots indicate the missing studies estimated by the trim-and-fill method.

**Figure 2.** Jitterplots displaying the distribution of propensity scores after six different matchings. Circle sizes indicate the assigned weights for group comparison regarding the treatment effect.
